# The Microbiota Metabolite–Joint Axis: Mechanistic Insights and Therapeutic Targets for Osteoarthritis

**DOI:** 10.3390/biomedicines14071566

**Published:** 2026-07-13

**Authors:** Gaoyu Song, Junwen Jing, Ziliang Su, Fan Wu, Xiaohua Chen, Jing Zhang, Feng He, Shibin Yu

**Affiliations:** State Key Laboratory of Oral & Maxillofacial Reconstruction and Regeneration, National Clinical Research Center for Oral Diseases, Shaanxi International Joint Research Center for Oral Diseases, Department of Oral Anatomy and Physiology, School of Stomatology, The Fourth Military Medical University, Xi’an 710032, China

**Keywords:** osteoarthritis, microbiota, metabolites, pathological mechanisms, ferroptosis

## Abstract

**Background**: Osteoarthritis (OA) is the most common degenerative joint disease globally, with its pathogenesis yet to be fully elucidated. Accumulating evidence has redefined OA as a systemic low-grade inflammatory disorder. While the gut microbiota–joint axis is widely recognized as a key regulatory pathway in OA progression, a definitive causal and mechanistic framework linking microbiota-derived metabolites to OA pathology has not yet been established. **Aim of Review**: This review aims to comprehensively evaluate the roles of microbiota-derived metabolites in OA pathogenesis by integrating cutting-edge multi-omics data, causal evidence from Mendelian randomization studies, and advanced translational strategies. We propose a conceptual framework linking microbial dysbiosis to joint degeneration and discuss potential therapeutic targets for OA. **Key Scientific Concepts of Review**: Microbiota-derived signals, including lipopolysaccharides, peptidoglycans, short-chain fatty acids, bile acids, tryptophan metabolites, and hydrogen sulfide, are associated with mucosal barrier impairment, aberrant immune activation, and metabolic–endocrine dysfunction. These systemic host responses, in turn, may collectively contribute to the three core pathological hallmarks of OA: cartilage degeneration, synovial inflammation, and subchondral bone remodeling. We highlight novel regulatory pathways, including bile acid–glucagon-like peptide-1(GLP-1) signaling, aryl hydrocarbon receptor (AhR) modulation, and ferroptosis regulation, as potential critical mediators of OA. Causal evidence from multi-omics and Mendelian randomization analyses is synthesized to move beyond simple descriptive associations. Furthermore, we discuss translational strategies, such as metabolite-targeted interventions (GUDCA, IPA, HDCA) and engineered bacterial extracellular vesicle delivery systems, providing a potential framework for the precision theranostics of OA.

## 1. Introduction

Osteoarthritis (OA) is the most prevalent degenerative joint disorder and a leading cause of global disability, substantially impacting patients’ quality of life. Data from the Global Burden of Disease (GBD) study indicate that the global prevalence of OA rose from 20.9 million to 46.63 million cases between 1990 and 2021 [1]. The World Health Organization estimates that over 500 million individuals worldwide are affected by OA, with most cases involving the knee, hip, hand joints, spine, or temporomandibular joint [2]. In China, the National Health Commission reports a prevalence rate of approximately 15%, corresponding to more than 150 million affected individuals [3]. The prevalence of OA increases sharply with age, affecting nearly 60–70% of individuals aged 65 years or older [4]. It thus represents a leading cause of functional disability among older adults worldwide.

The core pathological features of OA are progressive cartilage degeneration, chronic synovial inflammation, and aberrant subchondral bone remodeling [5,6]. Importantly, OA is now recognized as a whole-joint disease, as pathological changes occur not only in articular cartilage but also affect the synovium, subchondral bone, ligaments, periarticular muscles, and the infrapatellar fat pad. These tissues interact through complex biomechanical and biochemical crosstalk, contributing collectively to disease onset and progression. In addition to this multi-tissue involvement, OA is a highly heterogeneous disease encompassing multiple clinical phenotypes that differ in their predominant pathological features, progression patterns, and inflammatory profiles [7]. Recognizing this heterogeneity is crucial for understanding the differential contribution of the microbiota–joint axis, as factors such as obesity, metabolic syndrome, and post-traumatic etiology differentially modulate gut microbial composition and systemic inflammation.

Traditionally, OA has been attributed to mechanical and metabolic risk factors including aging, abnormal mechanical loading, and obesity [8]. However, these factors alone cannot fully account for the diverse pathological manifestations, systemic inflammatory profile, and heterogeneous disease progression observed in OA. The established contribution of systemic low-grade inflammation to OA pathogenesis suggests that additional extra-articular mechanisms may participate in disease initiation and progression. In this context, the recently proposed concept of the microbiota–joint axis offers new insights into OA pathogenesis. Accumulating correlational evidence from clinical cohorts shows that gut microbial dysbiosis is closely associated with OA onset and severity. Recently, Mendelian randomization (MR) studies have further provided causal genetic evidence for the role of specific gut microbial taxa and metabolites in OA risk, supporting the existence of a microbiota–joint regulatory axis [9,10]. As integral components of the human microbiome, these microbial communities not only maintain intestinal and oral mucosal homeostasis but also modulate distal joint physiology and pathology through circulating microbial components and bioactive metabolites [11]. Dysbiosis, characterized by reduced α-diversity, altered β-diversity, and disrupted microbial metabolic function, has been implicated in OA onset and progression through the systemic inflammatory cascade [12,13,14]. While the oral microbiota has also been implicated in OA—with reports of increased oral opportunistic pathogens such as *Porphyromonas, Streptococcus*, and *Fusobacterium* in OA patients—the mechanistic evidence remains preliminary and largely correlational [15]. Given the preponderance of mechanistic and causal evidence from the gut, this review focuses primarily on the gut microbiota–joint axis, with the oral–gut–joint axis acknowledged as an important direction for future investigation.

This narrative (mechanistic) review synthesizes current evidence linking microbiota-derived components and metabolites to the three core pathological processes of OA. By integrating systemic mechanisms with tissue-specific effects, we propose a mechanistic framework that bridges microbial dysbiosis and joint degeneration.

## 2. Pathways by Which Microbiota and Their Metabolites Regulate OA Progression

**Figure 1:**
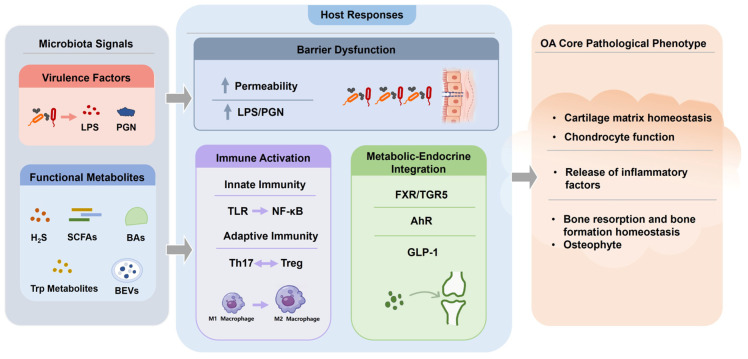
Microbiota Signals, Host Responses and Regulation of OA Pathological Outcomes. Abbreviations: AhR, aryl hydrocarbon receptor; BAs, bile acids; BEV, bacterial extracellular vesicles; FXR, farnesoid X receptor; GLP-1, glucagon-like peptide-1; H_2_S, hydrogen sulfide; LPS, lipopolysaccharide; NF-κB, nuclear factor-kappa B; OA, osteoarthritis; PGN, peptidoglycan; SCFAs, short-chain fatty acids; Th, T helper cell; TLR, Toll-like receptor; Treg, regulatory T cell; Trp, tryptophan.

The systemic pathogenesis of OA arises from a bidirectional interplay between microbiota-derived signals and coordinated multisystem host responses. Through the release of virulence factors and functional metabolites, the microbiota can influence long-distance pathological regulation. The host, in turn, may exerts either pathogenic or protective effects via cascading responses involving three principal systems: the mucosal barrier, the immune system, and the metabolic–endocrine system. An imbalance between microbial signals and host responses may represent an important contributing factor in the onset and progression of OA (as depicted in Figure 1).

### 2.1. Regulation by the Microbiota

The microbiota can modulate host physiology by releasing virulence factors and secreting functional metabolites that potentially influence OA progression.

#### 2.1.1. Direct Release and Transport of Endogenous Virulence Factors.

Under dysbiotic conditions, pathogenic bacteria undergo abnormal outgrowth and actively release structural components and virulence factors. These factors can translocate across tissues through a compromised mucosal barrier, potentially serving as initiating signals in OA pathogenesis. Most bacterial virulence factors are structural components of microorganisms, and their excessive release during dysbiosis confers considerable pathogenic potential. Bacterial cell wall components, such as lipopolysaccharide (LPS) and peptidoglycan (PGN), are continuously released following disruption of microbial homeostasis and subsequently enter the systemic circulation through an impaired intestinal or oral mucosa, leading to activation of host innate immune pathways [16,17].

Clinical studies have reported associations between elevated circulating LPS levels are closely associated with OA severity, including osteophyte formation, joint space narrowing, pain, and functional impairment. LPS is thought to promote OA pathology predominantly through activation of intra-articular macrophages and triggering of downstream NF-κB signaling [18,19]. Notably, serum LPS levels are significantly higher in patients with obesity-related OA, suggesting that gut barrier dysfunction (“leaky gut” syndrome) may contribute to systemic inflammation and OA pathogenesis via LPS translocation [20].

Animal studies provide further supporting evidence. In mouse models of OA induced by destabilization of the medial meniscus (DMM), germ-free mice exhibited significantly attenuated cartilage degeneration and osteophyte formation compared with conventionally housed controls. Moreover, serum levels of LPS and lipopolysaccharide-binding protein (LBP) were closely correlated with inflammatory disease progression, and serum LBP levels showed a positive association with the severity of cartilage degeneration [21].

Peptidoglycan (PGN), an arthritogenic component of bacterial cell walls, has been widely detected in synovial tissues from patients undergoing total knee arthroplasty, suggesting that PGN may contribute to the development of inflammatory synovitis [22]. Notably, *Micrococcus luteus* G18—a PGN-rich strain highly enriched in the synovial fluid of OA patients—has been identified as a key joint-resident pathogenic bacterium that is proposed to drive cartilage damage [23].

#### 2.1.2. Secretion of Functional Metabolites

The microbiota generates a diverse array of bioactive metabolites through multiple pathways, including fermentation of dietary fibers, amino acid metabolism, and transformation of host-derived primary bile acids. Primary examples include short-chain fatty acids (SCFAs), hydrogen sulfide (H_2_S), bile acids (BAs), and tryptophan (Trp) metabolites, etc. These metabolites can exert systemic effects by engaging specific host receptors, thereby modulating physiological homeostasis in distal joints. In this section, we provide a concise overview of each metabolite class, highlighting their sources and key functional findings, while reserving the detailed mechanistic dissection of their effects on cartilage, the synovium, and the subchondral bone for Section 3.

SCFAs, such as butyrate, acetate, and propionate, are the primary products of gut microbial fermentation of dietary fibers. Their biological effects are primarily mediated via G protein-coupled receptors (GPCRs, including GPR41, GPR43, and GPR109A) and inhibition of histone deacetylases (HDACs) [24]. SCFAs are considered important for maintaining intestinal barrier integrity, thereby helping to limit translocation of LPS into the circulation [25].

H_2_S is primarily the product of the microbial metabolism of sulfur-containing amino acids by sulfate-reducing bacteria. It has been shown to exerts diverse protective effects, including anti-inflammatory, antioxidant, and antiapoptotic actions [26,27,28,29].

Bile acids (BAs) are synthesized in the liver as primary bile acids and subsequently converted into secondary bile acids by the gut microbiota. Secondary bile acids modulate systemic energy metabolism, inflammatory responses, and bone homeostasis by activating the farnesoid X receptor (FXR) and Takeda G protein-coupled receptor 5 (TGR5) [30]. Recent studies have identified potential BA-mediated regulatory pathways in OA. Mulberry water extract has been shown to enrich *Lactobacillus johnsonii* in the gut, which promotes the generation of the secondary bile acid hyodeoxycholic acid (HDCA), thereby alleviating cartilage damage in OA mouse models [31]. Additionally, a landmark study reported that glycoursodeoxycholic acid (GUDCA), a secondary bile acid reduced in OA patients, attenuates cartilage degradation through intestinal FXR–GLP-1 signaling [32]. Consistently, population-based studies have linked ursodeoxycholic acid (UDCA) use to a lower risk of OA-related joint replacement [33]. The detailed molecular mechanisms of BA-mediated regulation in cartilage, synovium, and subchondral bone are discussed in Section 3.

Trp metabolites, generated through gut microbiota-mediated metabolism of dietary tryptophan, serve as key endogenous ligands for the aryl hydrocarbon receptor (AhR) [34]. AhR is widely expressed in immune cells and joint-resident cells, and its activation profoundly modulates inflammation and cellular metabolism [35]. Clinical studies in patients with erosive hand osteoarthritis (EHOA) have revealed altered gut microbiota composition and disrupted tryptophan metabolic profiles [36]. In experimental rat models, OA severity correlates positively with AhR pathway activation, and modulation of gut microbiota-dependent tryptophan metabolism has been shown to influence synovial inflammation [37].

Bacterial extracellular vesicles (BEVs) are lipid bilayer nanovesicles ranging from 20 to 400 nm in diameter, secreted by both Gram-negative and Gram-positive bacteria. Acting as a “long-range weapon” of bacteria, BEVs can deliver encapsulated bacterial regulatory molecules (including metabolites, LPS, and nucleic acids) to distal joint tissues. This delivery system not only enhances the bioavailability and stability of the original signaling molecules but also enables co-delivery of multiple bioactive compounds, potentially facilitating direct modulation of host cell signaling pathways. Through these mechanisms, BEVs may influence the inflammatory and metabolic status of the joint [38,39].

In addition to the well-characterized metabolite classes discussed above, several other microbiota-derived metabolites have recently emerged as regulators of OA pathogenesis through their effects on chondrocyte ferroptosis [40]. Urolithins, including Urolithin A (UA) and Urolithin B (UB), are bioactive microbial metabolites derived from the gut bacterial processing of ellagitannins—polyphenolic compounds abundantly present in pomegranates, strawberries, walnuts, and raspberries [41]. Capsiate (CAT), another gut microbiota-derived metabolite, is a capsaicin analog with reported anti-inflammatory and metabolic regulatory properties [42]. Notably, these three metabolites have been independently reported to inhibit chondrocyte ferroptosis in OA models, thereby attenuating cartilage degeneration. Their detailed mechanisms are elaborated in Section 3.1.2.

### 2.2. Host Response Mechanisms

This section describes the systemic host responses—mucosal barrier, innate/adaptive immunity, and metabolic–endocrine integration—that are proposed to link microbial signals to joint-specific pathologies detailed in Section 3.

#### 2.2.1. Mucosal Barrier Response

The intestinal and oral mucosal barriers provide the first line of physical and immune defense against translocation of microbial components. Under homeostatic conditions, intact epithelial tight junctions effectively prevent paracellular passage of virulence factors [43,44]. Dysbiosis-induced barrier impairment increases mucosal permeability, thereby creating a gateway for LPS, PGN, and other bacterial factors to enter the systemic circulation [16]. Mucosal epithelial cells detect these components through pattern recognition receptors such as Toll-like receptors (TLRs), triggering local inflammatory responses and initiating a self-perpetuating cycle of barrier disruption, systemic signal dissemination, and amplified inflammation [19]. Fecal metabolomic studies in obesity-related OA patients have shown that altered levels of propionate, indole metabolites, and other microbial products are closely associated with intestinal barrier dysfunction, providing clinical evidence linking mucosal barrier impairment to OA [45].

#### 2.2.2. Activation of Innate and Adaptive Immunity

Microbial signals engage both innate and adaptive immune pathways. In the innate immune response, microbial virulence factors activate pattern recognition receptors (PRRs) on immune cells and joint-resident cells, subsequently triggering downstream signaling cascades such as NF-κB and MAPK. This leads to the production of proinflammatory cytokines, including tumor necrosis factor-α (TNF-α) and interleukin-1β (IL-1β), thereby initiating systemic low-grade inflammation [46]. Notably, PGN and LPS can activate inflammatory and catabolic signaling pathways in chondrocytes from patients with OA through Toll-like receptor 2 (TLR2) [47]. These systemic innate immune activation signals reach various joint tissues via circulation, and further amplify pathological effects in local microenvironments such as the synovium and cartilage.

In addition to innate immune responses, adaptive immunity is subsequently engaged in OA pathogenesis. Although OA is not classically considered an autoimmune or infectious disease, dysregulation of adaptive immunity has emerged as a key feature of OA immunopathology [48]. It is characterized by an imbalance between effector T cells (Th1, Th2, Th17) and regulatory T cells (Tregs) [49,50,51,52]. These systemic immune changes can subsequently affect joint tissues, as detailed in Section 3.2.

Experimental evidence further supports a potential causal role for the gut microbiota in immune-mediated OA progression. Transplantation of gut microbiota from healthy donors into mice with destabilization of the medial meniscus (DMM)-induced OA reshaped the systemic immune profile, attenuated synovial inflammation, and reduced osteophyte formation in recipient animals [53]. These findings provide direct evidence that the microbiota can modulate OA pathogenesis through immune regulation.

#### 2.2.3. Systemic Integration of Metabolic–Endocrine Homeostasis

The metabolic–endocrine system is proposed to act as a systemic integrator and amplifier within the microbiota–joint axis. Rather than passively receiving metabolite signals from the gut, the metabolic–endocrine system actively integrates these inputs with the host’s energy status and hormonal balance, thereby altering systemic and joint metabolic and inflammatory states and modulating the progression of OA.

Microbial metabolites engage host metabolic regulatory networks by functioning as signaling ligands. For instance, microbiota-derived secondary bile acids and tryptophan metabolites can influence systemic metabolism and immune responses through nuclear receptor pathways such as FXR and AhR, linking gut microbial activity to peripheral tissue physiology [30,35,37,54].

Importantly, the microbiota–metabolic–endocrine axis provides a unifying pathophysiological framework for classical OA risk factors, such as obesity and metabolic syndrome. During dysbiosis, reduced SCFA production impairs the secretion of enteroendocrine hormones, including glucagon-like peptide-1 (GLP-1) and peptide YY (PYY), which are critical for maintaining glucose homeostasis and appetite regulation [25,55]. This dysregulation is associated with key features of metabolic–endocrine disorders, such as systemic insulin resistance and an imbalanced leptin-to-adiponectin ratio [56]. The resulting state of metabolic inflammation (elevated circulating free fatty acids and proinflammatory adipokines) establishes a systemic proinflammatory and procatabolic milieu. This environment not only directly damages joint tissues but also lowers the threshold for mechanical or inflammatory stress, thereby synergizing with immune responses triggered by microbial signals to accelerate OA onset and progression [14,57].

Thus, the metabolic–endocrine response may represent a central integrative process that converts discrete local microbial signals into a sustained systemic inflammatory state, ultimately contributing to joint pathogenesis.

This schematic illustrates the conceptual framework of the microbiota–metabolite–joint axis in osteoarthritis pathogenesis.

Left panel (Microbiota-derived signals): The gut microbiota actively releases (1) virulence factors including lipopolysaccharide (LPS) and peptidoglycan (PGN), and (2) functional metabolites such as short-chain fatty acids (SCFAs), hydrogen sulfide (H_2_S), bile acids (BAs), and tryptophan (Trp) metabolites. Bacterial extracellular vesicles (BEVs) serve as additional delivery vehicles for these microbial signals.

Middle panel (Host response systems): Microbial signals engage three interconnected host defense systems: (a) Mucosal barrier—intestinal and oral epithelial barriers that prevent systemic translocation of microbial products; (b) Immune system—activation of innate immunity (via TLRs/NF-κB) and adaptive immunity (Treg/Th17 balance); (c) Metabolic–endocrine system—integration of signals through enteroendocrine hormones (e.g., GLP-1), nuclear receptors (FXR, AhR), and systemic metabolic regulation.

**Figure 2:**
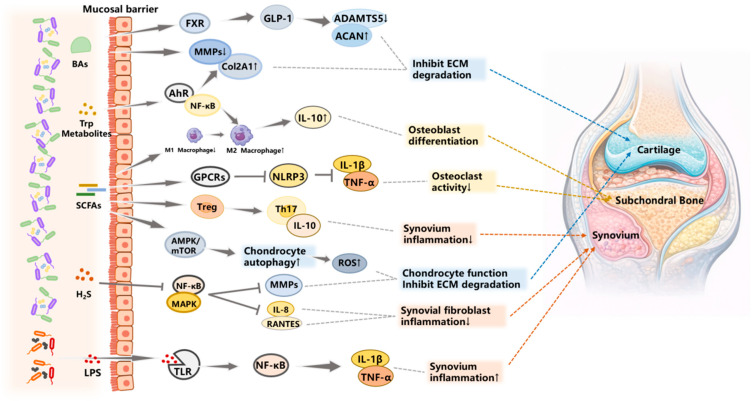
Key molecular mechanisms of representative microbiota-derived metabolites in regulating core OA pathological phenotypes. Symbol convention: → = activation/promotion/signal transduction; —| = inhibition/suppression; rectangular boxes = functional molecular or tissue modules. This diagram details tissue-specific signaling pathways of core microbial metabolites across cartilage, synovium and subchondral bone, expanding on the conceptual framework in Figure 1. Cartilage: Bile acids (BAs) inhibit extracellular matrix degradation via the intestinal FXR–GLP-1 axis, downregulating ADAMTS5 and upregulating ACAN and COL2A1. Tryptophan metabolites modulate matrix homeostasis and inflammation through the AhR–NF-κB pathway. Short-chain fatty acids (SCFAs) suppress MMP-mediated matrix degradation via GPCR and NF-κB signaling, and enhance chondrocyte autophagy via the AMPK/mTOR pathway. Hydrogen sulfide (H_2_S) inhibits NF-κB and MAPK cascades to reduce catabolic enzyme expression and maintain chondrocyte function. Synovium: Bacterial LPS activates TLR–NF-κB signaling to induce proinflammatory cytokine release. H_2_S suppresses synovial fibroblast inflammation via MAPK inhibition and modulates macrophage polarization. SCFAs restore the Th17/Treg balance to alleviate synovial inflammation. Subchondral bone: SCFAs inhibit osteoclast activity via GPCR-mediated NLRP3 inflammasome suppression. Tryptophan metabolites and SCFAs promote M2 macrophage polarization and IL-10 secretion to favor bone anabolism. BAs drive osteoblast differentiation via FXR activation. Abbreviations: ACAN, aggrecan; ADAMTS, a disintegrin and metalloproteinase with thrombospondin motifs; AhR, aryl hydrocarbon receptor; AMPK, AMP-activated protein kinase; BAs, bile acids; COL2A1, collagen type II alpha 1 chain; FXR, farnesoid X receptor; GLP-1, glucagon-like peptide-1; GPCRs, G protein-coupled receptors; H_2_S, hydrogen sulfide; LPS, lipopolysaccharide; MAPK, mitogen-activated protein kinase; MMP, matrix metalloproteinase; mTOR, mammalian target of rapamycin; NF-κB, nuclear factor-kappa B; NLRP3, NOD-like receptor thermal protein domain associated protein 3; SCFAs, short-chain fatty acids; Th, T helper cell; TLR, Toll-like receptor; Treg, regulatory T cell; Trp, tryptophan.

Right panel (OA pathological outcomes): The integrated host responses synergistically drive the three core pathological hallmarks of OA: (a) cartilage degeneration, characterized by disruption of cartilage matrix homeostasis and chondrocyte dysfunction; (b) synovial inflammation, marked by release of inflammatory factors; and (c) subchondral bone remodeling, involving an imbalance between bone resorption and bone formation, as well as osteophyte formation. (The detailed molecular mechanisms are depicted in Figure 2).

## 3. Mechanisms Underlying the Role of Microbiota and Their Metabolites in the Onset and Progression of OA

### 3.1. Mechanisms Underlying Microbiota-Mediated Cartilage Degeneration

Cartilage degeneration represents the central pathological hallmark of OA, characterized by ECM degradation (loss of type II collagen and proteoglycans) and chondrocyte dysfunction (metabolic disturbances, inhibited cell proliferation, and increased cell death). Accumulating evidence indicates that the gut microbiota and its metabolites influence cartilage homeostasis through both direct and indirect mechanisms.

#### 3.1.1. Inhibition of Cartilage Matrix Degradation

The gut microbiota and its metabolites can inhibit the activity of key enzymes responsible for ECM degradation, thereby delaying cartilage degeneration. In a spontaneous guinea pig model of OA, supplementation with *Bifidobacterium longum* CBi0703 alleviated cartilage damage by reducing type II collagen degradation [58]. Similarly, in a monosodium iodoacetate-induced rat model of OA, intervention with *Clostridium butyricum* suppressed the mRNA expression of multiple MMPs and tissue inhibitors of metalloproteinases (TIMPs), including MMP-2, MMP-3, MMP-9, MMP-13, TIMP-1, and TIMP-2 [59].

Butyrate, a well-studied microbiota-derived metabolite, has been shown to maintain cartilage homeostasis through multi-target mechanisms. Butyrate can significantly reduce IL-1β-induced upregulation of MMP-1, MMP-3, MMP-13, ADAMTS4, ADAMTS5, and ADAMTS9 at both the gene and protein levels. This occurs through multiple mechanistic pathways: inhibition of NF-κB signaling via blockade of IKK/IκBα/p65 phosphorylation [60], suppression of HDAC activity [61], and activation of the GPR43 receptor [62], collectively resulting in downregulation of inflammatory mediators and adhesion molecules. Consequently, butyrate reduces type II collagen degradation and preserves ECM integrity. Supplementation with the live *Lactobacillus* strain LA-1 improves the intestinal microenvironment and increases the abundance of butyrate-producing *Faecalibacterium* strains. Butyrate suppresses IL-1β-induced MMP-1 and MMP-13 expression via the AMPK/mTOR pathway, ultimately alleviating cartilage degeneration [63].

H_2_S reduces the synthesis and release of MMP-2, MMP-13, and ADAMTS5 by inhibiting the NF-κB and MAPK signaling pathways [64,65]. Administration of the H_2_S donor GYY4137 significantly decreases MMP-13 expression in joint tissues of DMM-induced OA mice, thereby effectively reducing type II collagen and proteoglycan degradation [66]. Similarly, another H_2_S donor, sodium hydrosulfide (NaHS), dose-dependently inhibits the overexpression of catabolic mediators (MMP-13, PGE_2_, and NO) in IL-1β-stimulated human OA chondrocytes. This effect is mediated by suppression of the extracellular signal-regulated kinase and NF-κB pathways, ultimately mitigating ECM degradation [67].

In addition to SCFAs and gaseous signaling molecules, microbiota-derived bile acid metabolites have also emerged as regulators of ECM homeostasis. As outlined in Section 2.1.2, glycoursodeoxycholic acid (GUDCA) inhibits intestinal FXR signaling and stimulates GLP-1 secretion; this gut-derived signal downregulates ADAMTS5 and upregulates aggrecan (ACAN) in cartilage, attenuating cartilage degradation. Ursodeoxycholic acid (UDCA), a clinically approved agent, has also shown chondroprotective effects in experimental OA models [32]. In DMM-induced OA mice, tauroursodeoxycholic acid (TUDCA) reduces MMP-13 levels in articular cartilage, thereby inhibiting collagen degradation, while concurrently increasing COL2A1 expression. This bidirectional regulation contributes to the maintenance of ECM homeostasis [68]. Trp metabolites, including indole-3-aldehyde (3-IAld) and indole-3-propionic acid (IPA), also exert protective effects on cartilage ECM by suppressing matrix-degrading enzymes (MMP-3, MMP-13, ADAMTS5) and promoting proteoglycan and type II collagen synthesis [69,70].

Collectively, these findings indicate that SCFAs (especially butyrate), H_2_S, and bile acids may protect cartilage matrix by suppressing MMP/ADAMTS expression through NF-κB-dependent and FXR-dependent pathways. Enhancing these metabolites via prebiotics, H_2_S donors, or bile acid analogs represents a promising strategy to slow cartilage degradation.

#### 3.1.2. Regulation of Chondrocyte Functional Homeostasis

Microbiota-derived metabolites, including butyrate, H_2_S, BAs and BEVs, regulate chondrocyte survival, metabolism, inflammatory status, and oxidative stress responses through both direct and indirect mechanisms, thereby maintaining cellular functional homeostasis.

Butyrate enhances chondrocyte autophagy by activating the AMPK/mTOR signaling pathway and reduces apoptosis by suppressing necroptosis- and apoptosis-related factors [63]. By enhancing autophagic clearance of damaged organelles, butyrate can significantly reduce reactive oxygen species (ROS) levels, prevent G1 phase cell cycle arrest, and modulate the expression of apoptosis-related proteins such as Bax and Bcl-2, thereby alleviating oxidative stress-induced injury [71,72]. Butyrate also downregulates multiple IL-1β-induced pro-inflammatory mediators in chondrocytes, including classic inflammatory factors (e.g., Nos2 and IL-6), proinflammatory adipokines (lipocalin-2 and nesfatin-1), and adhesion molecules (VCAM-1 and ICAM-1) [62].

H_2_S exerts multifaceted protective effects on chondrocytes. The H_2_S donor NaHS significantly reduces the expression of inducible nitric oxide synthase (iNOS) and IL-6 in osteoarthritic chondrocytes [73]. It also decreases caspase-3 activity in IL-1β-stimulated chondrocytes and reverses the aberrant expression of dynamin-related protein 1 (Drp1), a key regulator of mitochondrial fission, thereby inhibiting chondrocyte apoptosis [74]. S-propargyl-cysteine (SPRC), an endogenous H_2_S donor, attenuates IL-1β-induced COX-2 and MMP expression [75]. Diallyl disulfide (DADS), a garlic-derived H_2_S donor, scavenges ROS and reduces lipid peroxidation products (4-HNE and MDA) by activating the Nrf2/NQO1 pathway, while upregulating SOD and GPx activity [76]. Collectively, H_2_S maintains chondrocyte homeostasis through anti-inflammatory, anti-apoptotic, and antioxidant mechanisms.

Tauroursodeoxycholic acid (TUDCA) promotes the proliferation and differentiation of degenerative chondrocytes isolated from osteoarthritic knee joints. Mechanistically, TUDCA reduces intracellular cholesterol levels, increases membrane fluidity, and activates TGF-β receptor signaling and focal adhesion proteins. In DMM-induced mice, weekly intra-articular injection of 17.5 mM TUDCA significantly attenuated cartilage degeneration, achieving a nearly 40% reduction in Mankin scores [68].

AhR-mediated regulation directly impacts cartilage homeostasis []. Kynurenine, a tryptophan metabolite abnormally elevated in the synovial fluid of patients with OA, activates the AhR signaling pathway in human umbilical cord mesenchymal stromal cells (hUC-MSCs). Specifically, AhR activation in hUC-MSCs induces its nuclear translocation and upregulates the expression of its downstream target genes CYP1A1 and CYP1B1. This significantly inhibits the expression of SOX9, COL2A1, and ACAN and suppresses cell proliferation, ultimately impairing the chondrogenic capacity of hUC-MSCs. Conversely, AhR gene knockout in hUC-MSCs reverses these inhibitory effects and significantly enhances their chondroprotective function in rat OA models induced by anterior cruciate ligament transection (ACLT) and DMM [72]. In contrast, 3-IAld attenuates NF-κB pathway phosphorylation and p65 nuclear translocation by activating the AhR–NF-κB axis in chondrocytes, thereby inhibiting IL-6, iNOS, and COX-2 expression [69]. IPA exerts even more pronounced effects through the same pathway, reducing circulating inflammatory mediators and cartilage catabolic enzymes while increasing matrix synthesis in ACLT rats [70]. Thus, AhR activation yields opposing cartilage-regulating effects depending on ligand structure and cell context. This reflects biased agonism: different ligands induce distinct AhR conformations, leading to selective recruitment of co-activators or co-repressors [77]. This functional dichotomy underscores the need for selective AhR modulation in OA therapy.

Joint-resident microbiota can directly disrupt chondrocyte homeostasis. Micrococcus luteus G18, highly enriched in OA synovial fluid, releases peptidoglycan that activates the TLR2/JNK/AP-1 axis in chondrocytes, promoting apoptosis and inhibiting ECM synthesis [23].

Regulation of ferroptosis represents an additional mechanism. As introduced in Section 2.1.2, urolithins and capsiate (CAT) inhibit chondrocyte ferroptosis. Urolithin B (UB) upregulates FGFR3, disrupting the NCOA4–FTH1 interaction to suppress ferritinophagy and reduce labile iron accumulation; FGFR3 knockdown abolishes this protection [78]. Urolithin A (UA) activates the AMPK/mTOR/HIF-1α pathway, reducing MDA and Fe^2+^ while increasing GSH and GPX4 [79]. CAT inhibits ferroptosis via the SLC2A1/HIF-1α axis, and its levels negatively correlate with OARSI scores in OA mice [80]. These findings establish a framework wherein specific microbiota-derived metabolites converge on ferroptosis inhibition through distinct iron-regulatory and antioxidant pathways.

In summary, H_2_S and TUDCA may promote chondrocyte survival via anti-apoptotic and anti-ferroptotic pathways, while tryptophan metabolites can exert ligand-specific effects through AhR-biased agonism. Local delivery or gut-targeted strategies to elevate these metabolites may warrant clinical investigation.

### 3.2. Regulation of Synovial Inflammation

Synovial inflammation represents a core pathological feature of OA, characterized by synovial hyperplasia, infiltration of inflammatory cells, and excessive production of proinflammatory mediators. Accumulating evidence indicates that gut dysbiosis and microbiota-derived metabolites can either aggravate or alleviate synovial inflammation. While Section 2.2 described systemic immune responses to microbial signals, here we focus on the synovial tissue-specific pathological mechanisms.

#### 3.2.1. Induction of Proinflammatory Mediator Release

Clinical and population-based studies have demonstrated that alterations in gut microbial composition are closely associated with the severity of synovial inflammation in OA. The relative abundance of *Prevotella* is significantly increased in the gut microbiota of patients with OA and is positively correlated with serum inflammatory markers, including C-reactive protein (CRP) and IL-6. Similarly, an imbalanced proportion of *Bacteroides* is associated with the severity of synovitis [81]. Population-based studies further reveal that the relative abundance of *Prevotellaceae* and *Prevotella* in the gut is higher in individuals with hand synovitis than in those without [54]. Patients with symptomatic hand OA exhibit higher relative abundances of *Bilophila* and *Desulfovibrio* and a lower abundance of *Roseburia* in the gut [82]. These two proinflammatory genera are closely associated with chronic systemic inflammation [83,84].

In germ-free mice receiving fecal microbiota transplantation (FMT) from patients with knee osteoarthritis complicated with metabolic syndrome, synovitis was more severe following meniscal or ligamentous injury. Further analysis revealed significantly increased abundance of *Fusobacterium* and *Faecalibacterium* and decreased abundance of *Ruminococcaceae* in the gut of these mice. Alterations in the abundance of these three microbial taxa were positively correlated with the severity of synovitis and with plasma levels of IL-1β, IL-6, and macrophage inflammatory protein-1α (MIP-1α) in the mice [85]. Notably, *Faecalibacterium* has traditionally been regarded as a beneficial butyrate-producing genus with anti-inflammatory properties. Its positive correlation with synovitis severity thus presents an apparent paradox. This may be explained by strain-level functional heterogeneity within the genus and the disease-context-specific nature of the FMT donor population, highlighting that genus-level correlations should be interpreted cautiously and that strain-resolved metagenomic studies are needed.

Beyond these specific microbial associations, dysbiosis may promote chronic synovitis through general mechanisms involving microbial products such as LPS. As described in Section 2.1.1, LPS activates TLR4/NF-κB signaling; in the synovium, this leads to persistent upregulation of IL-1β and TNF-α, driving chronic synovitis [81]. Collectively, these findings indicate that abnormal expansion of specific microbial genera can exacerbate synovitis in OA by amplifying systemic and local joint inflammatory responses [86].

#### 3.2.2. Regulation of Immune Cell Polarization and Function

Microbiota-derived metabolites play a critical role in modulating the functional status of synovial macrophages and fibroblasts. The endogenous H_2_S donor SPRC attenuates synovial inflammation in temporomandibular joint osteoarthritis (TMJOA) by regulating macrophage polarization via inhibition of the JAK/STAT signaling pathway [75]. In vitro, SPRC supplementation markedly reduced the expression of M1 markers (CD86 and iNOS) and proinflammatory cytokines (TNF-α and IL-6) in LPS-stimulated RAW264.7 macrophages, while upregulating M2 markers (Arg-1 and IL-10). In vivo, SPRC administration decreased the infiltration of iNOS-positive M1 macrophages and increased the proportion of Arg-1-positive M2 macrophages in the synovium of TMJOA rats, thereby lowering synovitis scores and synovial lining thickness [75]. HDCA, a secondary bile acid produced by *Lactobacillus johnsonii*, also promotes synovial macrophage polarization toward the M2 phenotype, thereby alleviating synovial inflammation and cartilage damage in OA mice [31]. Supplementation with the H_2_S donor GYY4137 significantly downregulates the expression of NLRP3 and caspase-1 in the synovium of DMM-induced OA mice, thereby inhibiting synovial cell pyroptosis and alleviating synovial inflammation [65]. Furthermore, H_2_S can reduce the secretion of chemokines (e.g., IL-8 and RANTES) by suppressing the MAPK pathway (JNK and p38), thereby mitigating the inflammatory response of synovial fibroblasts [87].

#### 3.2.3. Regulation of T Cell Differentiation and Immune Homeostasis

Gut microbiota and their metabolites also modulate synovial inflammation by regulating T cell differentiation and maintaining immune homeostasis. In a rat model of OA induced by intra-articular injection of monosodium iodoacetate (MIA), oral administration of *Lactobacillus casei* combined with type II collagen and glucosamine more effectively inhibited the NF-κB signaling pathway in CD4^+^ T cells. This treatment reduced the secretion of IL-1β, TNF-α, and interferon-γ (IFN-γ), thereby decreasing inflammatory cell infiltration and synovial lining thickness [88]. Butyrate induces Treg cell differentiation by promoting histone acetylation and regulates the Th17/IL-10 immune balance, effectively alleviating synovial inflammation in collagen-induced arthritis [89,90]. Dietary propionate attenuates antigen-induced arthritis and monosodium urate crystal-induced arthritis. Propionate regulates adaptive immunity by skewing the Th1/Th17 phenotype toward a Treg-dominant profile and modulates innate immunity by suppressing NF-κB activation in myeloid cells, thereby reducing the expression of TNF-α and IL-1β. Furthermore, propionate directly inhibits the migration of synovial fibroblasts and decreases the expression of proinflammatory and destructive molecules such as C3 and RANKL, ultimately mitigating synovial inflammation and inflammatory tissue priming [91]. *Segmented filamentous bacteria* (SFB) drive autoantibody production and trigger synovial inflammation by inducing Th17 cell differentiation in the gut and promoting their subsequent migration to the spleen [92]. Collectively, these findings demonstrate that specific microbiota and their metabolites regulate OA-associated synovial inflammation by differentially controlling the homeostasis of T cell subsets, highlighting their potential as therapeutic targets for immune modulation.

Thus, mounting evidence supports that H_2_S and SCFAs reshape synovial immunity by promoting M2 macrophages and Tregs while suppressing M1 and Th17 cells. This supports the use of H_2_S donors, SCFA-boosting diets, and probiotics as adjunct therapies for OA synovitis.

### 3.3. Regulation of Subchondral Bone Remodeling

Subchondral bone remodeling represents a key pathological process in OA, characterized by disruption of the homeostatic balance between bone resorption and bone formation, accompanied by alterations in bone microarchitecture, mineral density, and biomechanical properties. Accumulating evidence indicates that this process is tightly regulated by systemic immune and metabolic pathways that are, in turn, shaped by the gut microbiota and its metabolites.

#### 3.3.1. Immune-Mediated Regulation of the Balance Between Bone Resorption and Bone Formation

SCFAs inhibit NLRP3 inflammasome activation by engaging GPR109A, thereby reducing the release of IL-1β and TNF-α and subsequently suppressing osteoclast activity [93]. Propionate and butyrate significantly attenuate wear particle-induced osteolysis by inhibiting NLRP3 inflammasome activation and macrophage pyroptosis. These SCFAs also downregulate osteoclast differentiation markers, including NFATc1, Oc-STAMP, and Oscar, as well as upstream regulators such as TRAF2, TRAF6, and c-Fos [94]. Butyrate also inhibits Th17 cell differentiation and reduces osteoclast generation, thereby suppressing subchondral bone resorption [95].

Water-soluble small molecules secreted by *Lactobacillus reuteri* 6475 act directly on CD4^+^ T cells in mesenteric lymph nodes. By inhibiting the RIP2/NOD signaling pathway, these components enhance IL-10 secretion and promote the expression of osteogenic factors such as osterix, thereby regulating subchondral bone remodeling through the gut–immune–bone axis [96]. *Lactobacillus acidophilus* similarly promotes Treg cell proliferation while suppressing the differentiation of osteoclast-promoting Th17 cells. This immunomodulatory shift is accompanied by downregulation of osteoclastogenic factors (IL-6, IL-17, TNF-α, and RANKL) and upregulation of anti-osteoclastic cytokines (IL-10 and IFN-γ). These changes ultimately reduce bone resorption, improve trabecular and cortical bone microarchitecture, and enhance bone mineral density (BMD) and bone heterogeneity in mice [97]. *Saccharomyces boulardii* CNCM I-745 blocks the TLR2/MYD88/NF-κB inflammatory pathway and decreases levels of proinflammatory cytokines such as IL-6 and TNF-α. This inhibition suppresses osteoclast activation and RANKL expression, thereby restoring bone remodeling balance in rats [98]. These findings indicate that the microbiota and their metabolites participate in bone resorption and formation through immunomodulatory mechanisms.

#### 3.3.2. Macrophage Phenotype Modulation in the Restoration of Bone Remodeling Homeostasis

The H_2_S donor GYY4137 promotes M2 polarization of BMDMs while suppressing the M1 phenotype. Co-culture of GYY4137-treated macrophages with MC3T3-E1 preosteoblasts enhanced cell viability and proliferation, promoted matrix mineralization, and upregulated BMP-2 and osterix [99]. SCFAs also modulate the macrophage phenotype systemically: gold nanoparticle intervention increases beneficial gut bacteria (e.g., *Akkermansia*, *Lactobacillus*) and SCFA levels, promoting M2 polarization and IL-10 secretion, which protects subchondral bone in ACLT-induced OA [100].

#### 3.3.3. Enhancing Osteogenesis via Microbiota-Metabolite-Nuclear Receptor Signaling

Gut microbiota-derived bile acids influence bone remodeling through nuclear receptors. Chenodeoxycholic acid (CDCA) activates FXR in human bone marrow stromal cells (hBMSCs), upregulating bone sialoprotein (BSP), osteocalcin (OC), osteopontin (OPN), and alkaline phosphatase (ALP). Silencing of FXR using short hairpin RNA (shRNA) completely abolishes these inductive effects, confirming the essential role of FXR signaling in CDCA-mediated osteogenesis [101]. Tryptophan metabolites indole-3-acetic acid (IAA) and indole-3-propionic acid (IPA) exert osteoprotective effects through intestinal AhR activation, which restores barrier integrity and induces M2 macrophages to secrete IL-10. Circulating IL-10 then promotes osteoblast differentiation and inhibits osteoclast formation, ameliorating bone loss [102].

#### 3.3.4. Regulation of Osteophyte Formation

Osteophyte formation is a hallmark of end-stage OA and may be linked to gut dysbiosis and altered metabolite profiles. Zhu et al. found that the SCFA-producing genus Blautia was more abundant in KOA patients with severe osteophytes, accompanying with increased serum level of leukotriene B4(LTB4) and prostaglandin D2(PGD2) [103]. While SCFAs are generally anti-inflammatory, this correlation may reflect SCFA-independent mechanisms, such as modulation of lipid mediators. PGD2 has been detected in OA synovial fluid [104], and subchondral osteoblasts in OA can synthesize LTB4, supporting a role in osteophyte formation [105]. Thus, gut microbiota may influence osteophyte formation through modulation of lipid mediators like LTB4 and PGD2.

Taken together, these results imply that SCFAs suppress osteoclast-driven bone resorption, while bile acids (CDCA) promote osteoblast formation via FXR. Modulating these metabolites could restore subchondral bone remodeling balance in OA.

### 3.4. Crosstalk Between Cartilage, Synovium, and Subchondral Bone: Modulation by Microbiota-Derived Metabolites

The three core pathological hallmarks of OA do not develop independently; extensive crosstalk between tissues drives disease progression. The synovium, cartilage, and subchondral bone form a functional unit, where pathological changes in one tissue trigger secondary damage in the others. Microbiota-derived metabolites can simultaneously modulate this inter-tissue crosstalk via shared signaling pathways, exerting holistic regulatory effects on OA progression. This systemic action distinguishes microbiota-targeted therapies from single-tissue local interventions.

Activated synovial macrophages and fibroblasts secrete proinflammatory cytokines (TNF-α, IL-1β) and matrix-degrading enzymes that diffuse into cartilage and subchondral bone, directly inducing chondrocyte apoptosis and osteoclast activation [5,6]. Degraded cartilage matrix fragments in turn amplify synovial inflammation and hyperplasia. Sclerotic subchondral bone secretes elevated levels of proinflammatory and angiogenic factors that exacerbate cartilage degeneration and synovitis. Metabolites such as butyrate, secondary bile acids, and tryptophan metabolites can simultaneously suppress pro-catabolic and pro-inflammatory pathways across all three tissue compartments, breaking this pathological positive feedback loop [51,60,65,70,93,102].

In addition to the cartilage, synovium, and subchondral bone, the infrapatellar fat pad (IPFP)—also known as Hoffa’s fat pad—has emerged as an important contributor to knee OA pathogenesis. The IPFP is the largest adipose tissue within the knee joint, composed of adipocytes, immune cells, and blood vessels [106]. Historically viewed as a mere structural cushion, it is now recognized as a metabolically active endocrine organ that secretes adipokines (e.g., adiponectin, leptin, TNF-α), pro-inflammatory cytokines (e.g., IL-6), and catabolic mediators [107]. These factors can contribute to synovial inflammation, cartilage degradation, and subchondral bone remodeling [108]. Reciprocally, inflammatory signals from the synovium and cartilage can stimulate IPFP fibrosis and hyperplasia [106], establishing a vicious cycle that parallels the cartilage–synovium–bone crosstalk described above. Importantly, the IPFP’s responsiveness to systemic metabolic and inflammatory signals—potentially including those originating from gut microbial dysbiosis—positions it as a plausible downstream effector of the microbiota–joint axis. Although direct evidence linking gut microbiota-derived metabolites to IPFP function remains limited, the IPFP’s responsiveness to systemic metabolic and inflammatory signals positions it as a plausible downstream effector of the microbiota–joint axis. Future studies investigating the IPFP as a mediator of this axis are warranted.

Currently, evidence from clinical cohorts and animal studies remains largely correlative. Conventional 2D cell culture and single-tissue in vitro models fail to recapitulate the dynamic multi-tissue crosstalk of the joint, while animal models suffer from interspecies differences that limit translational validity. This critical gap in causal validation can be addressed by advanced human-relevant in vitro platforms, which are discussed in detail in Section 4.2.4.

Criteria for translational potential grading: High—Interventions with an FDA-approved drug or readily translatable dietary/lifestyle modification already available for human testing (e.g., UDCA, dietary fiber). Medium—Robust preclinical evidence exists but requires further validation, dose optimization, or clinical trial initiation (e.g., probiotics, H_2_S donors, IPA). Low—Mechanistically promising but faces substantial barriers including lack of defined pharmacokinetics, biosafety concerns, or absence of scalable delivery systems (e.g., BEVs, CAT).

## 4. Conclusions and Outlook

In summary, gut dysbiosis and its derived metabolites are proposed to contribute to the pathological progression of OA via a triple interactive network that disrupts mucosal barrier function, induces aberrant innate and adaptive immune activation, and disturbs metabolic and endocrine homeostasis. These microbial signals and host systemic responses further synergistically regulate the three core pathological hallmarks of OA: articular cartilage degeneration, synovial inflammation, and abnormal subchondral bone remodeling. We outline the bidirectional regulatory roles of key microbiota-derived metabolites, including SCFAs, secondary BAs, Trp metabolites, and H_2_S, as well as the newly identified ferroptosis regulatory mechanism implicated in microbial metabolites in OA progression. Collectively, these observations suggest that the microbiota–joint axis acts as a central regulator of OA pathogenesis, prompting a shift in the understanding of OA from a local mechanical degenerative disease to a systemic metabolic inflammatory disorder.

### 4.1. Challenges in Clinical Translation

Although targeting microbiota-derived metabolites holds broad therapeutic promise for OA, its clinical translation still faces substantial challenges. OA is a highly heterogeneous disease encompassing multiple clinical phenotypes—including metabolic OA, post-traumatic OA, age-related OA, and erosive hand OA—that differ in their predominant pathological features, progression patterns, and inflammatory profiles. The role of the microbiota–joint axis likely varies across these phenotypes, as factors such as obesity, metabolic syndrome, and post-traumatic etiology differentially modulate gut microbial composition and systemic inflammation [109]. Several major hurdles must be overcome. First, inter-individual microbiome variability—influenced by genetic background, dietary patterns, age, comorbidities, and medication use—leads to inconsistent findings across clinical cohorts and variable responses to microbiota-targeted interventions, posing a challenge for developing universal or precision strategies such as customized prebiotic/probiotic formulations or appropriate donor selection for fecal microbiota transplantation. Second, validated microbial biomarkers for patient stratification are currently lacking, making it difficult to predict which OA endotypes will benefit from specific microbiome-modulating therapies. Third, delivery of bacterial extracellular vesicles (BEVs) to deep joint tissues (e.g., cartilage and subchondral bone) while avoiding systemic off-target effects remains a biotechnological hurdle. Fourth, most current clinical evidence derives from observational correlational studies, and high-quality causal evidence in human populations is still limited; pathological mechanisms verified in animal models have not been fully translated to humans, as highlighted by inconsistent findings in naturally occurring canine OA models. Moreover, the spatiotemporal dynamics of metabolite-mediated regulation of subchondral bone remodeling and osteophyte formation in human OA remain unclear, and the relative contributions of systemic gut microbiota signals versus local joint-resident microbial components have not been clearly distinguished. Overcoming these challenges will require large-scale longitudinal multi-omics studies, development of standardized microbial biomarkers, advanced bioengineering solutions for joint-specific delivery, and randomized controlled trials with rigorous stratification.

### 4.2. Future Directions

Future researchers should prioritize the following four directions:

#### 4.2.1. Multi-Omics Integration

Efforts to integrate metagenomics, metabolomics, and transcriptomics (extendable to single-cell transcriptomics) may help map the OA-specific “microbiota–metabolite–joint cell” interaction network. Jiang et al. provided an illustrative paradigm for temporomandibular disorder research by adopting a multi-omics strategy combining Mendelian randomization (MR) analysis, metabolomics, and transcriptomics, which helped clarify the potential causal relationship between gut microbiota and TMD and identify key downstream targets (e.g., the AGE-RAGE signaling pathway, genes related to cell adhesion and inflammation) [110]. Recent multi-omics studies from two Chinese OA cohorts have identified a link between gut microbial leucine metabolism and knee synovitis via the TWEAK signaling pathway [111], and multi-cohort studies of hand OA have revealed the critical role of dysregulated tryptophan metabolism in disease progression [112,113], providing a solid foundation for this analytical strategy. This framework of “causal verification-mediation analysis-target mining” can be directly applied to OA research, particularly to dissect how microbiota-derived metabolites (e.g., SCFAs, BAs) regulate chondrocyte dysfunction, synovial inflammation, and subchondral bone remodeling through specific molecular networks. To further improve the efficiency of multi-omics data integration, Shao et al. emphasized the potential of machine learning and deep learning methods: these computational tools can effectively integrate heterogeneous multi-omics datasets, reveal context-specific host–microbe interactions in OA, and provide a computational basis for constructing individualized interaction networks and guiding subsequent precision interventions [114]. Meanwhile, two-sample MR analysis based on large-scale genome-wide association study data should be widely used to verify the causal relationships between specific microbial taxa/metabolites and human OA, addressing the core limitation of current correlational studies.

#### 4.2.2. Engineering Strategies

Based on mechanisms revealed by multi-omics analyses, intelligent bacterial extracellular vesicle (BEV) delivery systems can be designed to address the low intra-articular bioavailability and systemic off-target effects of microbiota-derived metabolites. For example, modifying BEVs with joint tissue-targeting ligands (such as chondrocyte-specific antibodies or synoviocyte-binding peptides) may enable controlled, site-specific release of therapeutic metabolites (e.g., butyrate, indole-3-propionic acid, hyodeoxycholic acid, capsiate) in the joint cavity. This approach could potentially maximize chondroprotective, anti-inflammatory, and anti-ferroptotic effects while reducing systemic side effects. Nevertheless, the clinical translation of intra-articular BEV-based therapies requires rigorous consideration of biosafety risks. First, despite reduced immunogenicity relative to intact bacteria, BEV membranes retain inherent pathogen-associated molecular patterns (e.g., lipopolysaccharides, peptidoglycans) that may trigger local synovial inflammation or systemic innate immune activation upon repeated intra-articular injection [115]. engineered surface modifications may also introduce exogenous antigenic epitopes that further elevate immunogenic risks. Second, the long-term biocompatibility of locally retained BEVs within the closed joint cavity, as well as dose-dependent local cytotoxicity from sustained cargo release, remains to be systematically validated. Third, off-target systemic distribution of a fraction of BEVs may lead to gradual accumulation in reticuloendothelial organs (liver, spleen) over repeated dosing, with potential risks of long-term systemic toxicity and unintended disruption of endogenous gut microbiota homeostasis. To mitigate these concerns, future development should integrate genetic engineering to eliminate bacterial virulence factors, optimized purification protocols to minimize endotoxic residues, and comprehensive preclinical toxicological assessments, to balance therapeutic efficacy with clinical safety. In addition, oral colon-targeted delivery systems can be developed to improve the stability and targeted release of prebiotics and metabolite precursors in the gut, enhancing the in vivo efficacy of microbiota-targeted interventions.

#### 4.2.3. Clinical Validation and Translational Stratification

Translate the aforementioned mechanistic and technological advances into clinical practice by conducting large-scale, multi-center phase I/II clinical trials targeting key microbiota-associated pathways in OA. Specifically, trials can evaluate the safety and efficacy of FXR agonists—such as GUDCA or UDCA, a clinically approved drug with well-established safety—in promoting proteoglycan synthesis and delaying cartilage degeneration [32]. Alternatively, trials could assess the effectiveness of aryl hydrocarbon receptor (AhR) modulators (e.g., IPA) in inhibiting matrix metalloproteinase (MMP) expression and alleviating synovial inflammation [70]. Meanwhile, the efficacy of probiotics, prebiotics, and nutritional interventions (e.g., Mediterranean diet rich in fiber and polyphenols) should be systematically evaluated for different OA subtypes, including post-traumatic OA, metabolic syndrome-associated OA, and erosive hand OA [116]. Outcome measures should include not only changes in joint pain scores and physical function scales but also objective imaging indicators (cartilage thickness and subchondral bone microstructure measured by MRI), and serum/synovial fluid inflammatory and metabolic biomarkers, which can comprehensively reflect the therapeutic effect on joint structure and systemic inflammatory status.

To provide a clinically oriented perspective on the diverse strategies discussed throughout this review, we have graded their translational maturity in Table 1 (High/Medium/Low). Briefly, strategies graded as High include UDCA/GUDCA, which leverage an established safety profile and offer a direct pathway to OA trials, alongside dietary interventions (e.g., Mediterranean diet) supported by observational data. Strategies graded as Medium—probiotics, prebiotics, and FMT—show consistent preclinical efficacy but human data remain heterogeneous and inconsistent. Strategies graded as Low—BEVs, H_2_S donors, and ferroptosis-targeting metabolites (e.g., CAT)—are mechanistically innovative but lack defined pharmacokinetics, safety, and delivery systems for human translation. This stratification clarifies that while the mechanistic landscape is extensive, current clinical validity is largely confined to repurposing established agents and lifestyle modifications, whereas most emerging bioengineering and metabolite-based strategies require rigorous preclinical optimization prior to clinical consideration.

#### 4.2.4. Functional Validation Using Advanced In Vitro Models

As outlined in Section 3.4, existing cellular and animal models cannot fully replicate the multi-tissue interactive microenvironment of the human joint, nor can they directly establish causal links between microbiota-derived metabolites and inter-tissue pathological crosstalk. Organoids and organs-on-chips (OoCs) represent next-generation human-relevant platforms that fill this critical gap [121,122]. Joint-on-a-chip (JoC) systems address this gap. They recapitulate the dynamic interplay between cartilage, synovium, and subchondral bone. They incorporate physiologically relevant mechanical loading and fluid flow. They also support integrated co-culture of multiple cell types [122,123]. Modular platforms have combined synovial membrane-on-chip and cartilage-on-chip. These systems enable study of cross-tissue communication via inflammatory mediators [124]. Cartilage-on-a-chip models have reproduced OA-relevant phenotypes under hyperphysiological compression [125]. More recent microfluidic co-culture systems incorporate osteoblasts, chondrocytes, fibroblasts, and M1 macrophages [126]. Vascularized osteochondral chips have replicated the signaling axis between cartilage and subchondral bone [127].

Advances in gut-on-a-chip technology now support long-term co-culture of human intestinal epithelium with complex gut microbiota [128]. Coupling a gut-on-a-chip with a JoC could enable direct causal testing of key hypotheses, such as whether SCFAs regulate synovial macrophage polarization, whether secondary bile acids traverse the gut barrier to protect cartilage, and whether tryptophan metabolites activate AhR signaling and suppress MMP expression in real time. Such coupled platforms could permit continuous monitoring of metabolite transport and tissue responses, helping to transform the microbiota–joint axis from a correlative concept into a mechanistically testable framework.

The integration of artificial intelligence with organ-on-chip platforms promises to accelerate high-content image analysis, predictive modeling of metabolite–tissue interactions, and automated culture optimization [129]. Recent FDA initiatives endorsing human-relevant platforms as alternatives to animal testing further underscore the translational potential of these technologies for OA precision medicine [130].

Future efforts should prioritize standardized, multi-organ microphysiological systems. These systems should incorporate patient-specific microbial and cellular components. Such components could be derived from OA endotypes defined by multi-omics. This approach will help deconstruct the causal web of the microbiota–joint axis. It will also provide a high-fidelity screening platform for next-generation therapeutics.

Addressing these challenges may promote the translation of the “microbiota–joint axis” concept into clinical practice, facilitating the development of novel microbiota-based precision strategies for OA.

## Figures and Tables

**Table 1:** Microbiota and Metabolites Implicated in OA: Proposed Sources, Targets, Roles, and Summary of Evidence.

Metabolite/Microbiota	Source	Primary Targets/Pathways	Role in OA Pathogenesis	Key Experimental Evidence & Models (Type of Evidence)	Translational Potential
I. Specific Microbiota					
Proinflammatory Microbiota (e.g., *Prevotella*, *Bilophila*, *Desulfovibrio, Micrococcus luteus*)	Gut Microbiota	TLR4, NF-κB pathway	Associated with exacerbated synovitis; correlate with serum CRP/IL-6; Promotes chondrocyte apoptosis; inhibits ECM synthesis	Human cohort studies; FMT from OA patients aggravates mouse synovitis [81,82,83,84,85]; Highly enriched in OA synovial fluid [23]. (Observational + Preclinical)	Low (potential as biomarkers; causality unestablished)
Beneficial Microbiota (e.g., *Bifidobacterium*, *Clostridium*, *Lactobacillus*)	Gut Microbiota	SCFA production, immune modulation	May attenuate cartilage degeneration and regulate bone remodeling	*B. longum* ↓ cartilage degeneration [58]; *C. butyricum* ↓ MMPs [59]; *L. casei* & *L. acidophilus* modulate T cells & bone remodeling [88,97]. (Preclinical)	Medium (preclinical efficacy consistent; human data still limited)
II. Microbiota-Derived Metabolites					
LPS/PGN	Bacterial Cell Wall Components	TLR4 (LPS), TLR2 (PGN), NF-κB	Disrupt mucosal barrier; induce pro-inflammatory cytokines	LPS levels correlate with OA severity [18]; PGN upregulates MMPs in synovial fibroblasts [117]; PGN hydrolysis by *E. faecium* restores barrier [118]. (Human cohort + Preclinical)	Low (targeting LPS/TLR4 is challenging)
SCFAs (e.g., butyrate, propionate)	Gut microbial fermentation of dietary fiber	GPR41/43, HDAC, AMPK/mTOR, NF-κB/MAPK	↓ MMPs/ADAMTSs; ↑ collagen II; regulate Treg/Th17 balance	*C. butyricum* ↓ MMPs, ↑ TIMPs [59]. -Butyrate ↑ autophagy, ↓ apoptosis in chondrocytes [62,71]. Propionate ↓ synovial fibroblast migration [91]. Human RCTs: butyrate sustained-release tablets improved immune balance in hand OA (*n* = 33) [119]; butyrate capsules ↓ pain/stiffness and improved function in knee OA (*n* = 132) [120]. (Human RCT + Preclinical)	High (dietary intervention easily translatable)
BAs (e.g., UDCA, GUDCA, TUDCA, CDCA)	Hepatic synthesis+ gut microbial transformation	FXR, TGR5	↓ ADAMTS5 via intestinal FXR/GLP-1 axis; ↑ aggrecan/collagen II; promote osteogenesis via FXR	GUDCA delays cartilage degradation [32]; TUDCA ↑ chondrogenesis, ↓ OA progression [68]; CDCA ↑ osteoblast markers in hBMSCs [101]. Human cohort: UDCA users had lower risk of OA-related joint replacement (*n* = 5972) [32]. (Human cohort + Preclinical)	High (UDCA already FDA-approved; ready for repurposing trials)
H_2_S	Sulfate-reducing bacteria; protein fermentation	Nrf2/HO-1, Nrf2/NQO1, NF-κB/MAPK, JAK/STAT, Drp1 (mitochondrial fission)	Anti-inflammatory, anti-apoptotic, and antioxidant; ↓ MMP-2/MMP-13/ADAMTS5; ↑ M2 macrophage polarization; enhance osteogenesis via BMP-2/osterix	Cartilage: NaHS & GYY4137 ↓ catabolic factors & cartilage degradation [66,67]; NaHS ↓ caspase-3, reverses Drp1, inhibits mitochondrial apoptosis [74]; NaHS ↓ iNOS, IL-6 in OA chondrocytes [73]; DADS scavenges ROS, ↓ 4-HNE/MDA, ↑ SOD/GPx via Nrf2/NQO1 [76] Synovium/Bone: SPRC attenuates TMJOA synovitis via M2 polarization [75]; GYY4137 enhances osteoblast activity, ↑ BMP-2/osterix via M2 polarization [99]. (Preclinical)	Medium (H_2_S donors in early preclinical development)
Trp Metabolites (e.g., IPA, Kynurenine, 3-IAld, IAA)	Gut microbial metabolism of tryptophan	AhR	IPA/3-IAld, ↓ NF-κB, ↓ MMP-3/MMP-13/ADAMTS5, ↑ collagen II/proteoglycan; IAA/IPA ↑ osteogenesis, ↓ osteoclasts via IL-10. Kynurenine ↑ CYP1A1/CYP1B1, ↓ SOX9/COL2A1, impairs MSC chondroprotection	Protective: IPA ↓ IL-6, MMP-13 in ACLT rat model [70]; 3-IAld exerts anti-inflammatory & anabolic effects in chondrocytes [69]; IAA/IPA ameliorate bone loss in OVX mice [102]. Pathological: Kynurenine impairs chondroprotective function of MSCs [72]; (Preclinical)	Medium (IPA/3-IAld promising but requires further validation)
BEVs	Secreted by gut microbiota	TLR2/4, NF-κB, MAPK	Dual role: deliver anti-inflammatory cargo (e.g., SCFAs) or pro-inflammatory PAMPs (LPS, PGN); modulate innate immunity	*Lactobacillus*-derived BEVs ↓ osteoclastogenesis [39]; deliver SCFAs/Trp metabolites [38]. (Conceptual/proof-of-concept)	Low (significant biosafety and manufacturing hurdles)
Urolithins (UA, UB)	Gut microbial metabolism of ellagitannins (pomegranates, strawberries, walnuts)	UA: AMPK/mTOR/HIF-1α; UB: FGFR3/NCOA4/FTH1 axis (ferritinophagy inhibition)	Inhibit chondrocyte ferroptosis: ↓ MDA, Fe^2+^; ↑ GSH, GPX4; preserve ECM integrity	UA: AMPK inhibitor reverses protection in IL-1β-treated chondrocytes [79]; UB: FGFR3 knockdown abolishes anti-ferroptotic effects in ACLT rat model [78]. (Preclinical)	Low-Medium (natural product; oral bioavailability requires optimization)
Capsiate (CAT)	Gut microbial metabolite (capsaicin analog)	SLC2A1/HIF-1α	Inhibits ferroptosis-dependent cartilage damage; ↓ Fe^2+^ accumulation and lipid peroxidation via SLC2A1/HIF-1α	CAT levels negatively correlate with OARSI scores in OA mice; SLC2A1 silencing eliminates protection [80]. (Preclinical)	Low (requires further validation of in vivo delivery and safety)

Table footnotes: ↓ indicates reduction/inhibition; ↑ indicates increase/promotion. ADAMTS, a disintegrin and metalloproteinase with thrombospondin motifs; AhR, aryl hydrocarbon receptor; BA, bile acid; BEV, bacterial extracellular vesicle; CDCA, chenodeoxycholic acid; ECM, extracellular matrix; FMT, fecal microbiota transplantation; FXR, farnesoid X receptor; GUDCA, glycoursodeoxycholic acid; HDAC, histone deacetylase; H_2_S, hydrogen sulfide; IPA, indole-3-propionic acid; LPS, lipopolysaccharide; MMP, matrix metalloproteinase; NF-κB, nuclear factor-kappa B; OA, osteoarthritis; OARSI, Osteoarthritis Research Society International; PGN, peptidoglycan; SCFA, short-chain fatty acid; TGR5, Takeda G protein-coupled receptor 5; TLR, Toll-like receptor; Trp, tryptophan; TUDCA, tauroursodeoxycholic acid; UDCA, ursodeoxycholic acid; 3-IAld, indole-3-aldehyde.

## Data Availability

No new data were created or analyzed in this study.Data sharing is not applicable to this article.

## Abbreviations

OA: osteoarthritis; GBD: global burden of disease; LPS: lipopolysaccharide; PGN: peptidoglycan; LBP: lipopolysaccharide-binding protein; SCFAs: short-chain fatty acids; H_2_S: hydrogen sulfide; BAs: bile acids; Trp: tryptophan; GPCRs: G protein-coupled receptors; HDACs: histone deacetylases; MMPs: matrix metalloproteinases; ECM: extracellular matrix; mTOR: mammalian target of rapamycin; AMPK: AMP-activated protein kinase; NF-κB: nuclear factor-kappa B; CAT: capsiate; SLC2A1: solute carrier family 2 member 1; HIF-1α: hypoxia-inducible factor 1α; Nrf2: nuclear factor erythroid 2-related factor 2; HO-1: heme oxygenase-1; FXR: farnesoid X receptor; TGR5: Takeda G protein-coupled receptor 5; GLP-1: glucagon-like peptide-1; ADAMTS: a disintegrin and metalloproteinase with thrombospondin motifs; ACAN: aggrecan; UDCA: ursodeoxycholic acid; GUDCA: glycoursodeoxycholic acid; TUDCA: tauroursodeoxycholic acid; HDCA: hyodeoxycholic acid; CDCA: chenodeoxycholic acid; AhR: aryl hydrocarbon receptor; EHOA: erosive hand osteoarthritis; BEVs: bacterial extracellular vesicles; TLRs: Toll-like receptors; TNF-α: tumor necrosis factor-α; IL-1β: interleukin-1β; TLR2: Toll-like receptor 2; Th: T helper; Tregs: regulatory T cells; PYY: peptide YY; TIMPs: tissue inhibitors of metalloproteinases; COX-2: cyclooxygenase-2; 4-HNE: 4-hydroxynonenal; MDA: malondialdehyde; SOD: superoxide dismutase; GPx: glutathione peroxidase; TGF-β: transforming growth factor-β; hUC-MSCs: human umbilical cord mesenchymal stromal cells; iNOS: inducible nitric oxide synthase; IPA: indole-3-propionic acid; 3-IAld: indole-3-aldehyde; SPRC: S-propargyl-cysteine; DADS: diallyl disulfide; TMJOA: temporomandibular joint osteoarthritis; JAK: Janus kinase; STAT: signal transducer and activator of transcription; BMDMs: bone marrow-derived macrophages; BMP-2: bone morphogenetic protein-2; hBMSCs: human bone marrow stromal cells; BSP: bone sialoprotein; OC: osteocalcin; OPN: osteopontin; ALP: alkaline phosphatase; shRNA: short hairpin RNA; IAA: indole-3-acetic acid; KOA: knee osteoarthritis; LTB4: leukotriene B4; PGD2: prostaglandin D2; MR: Mendelian randomization; DMM: destabilization of the medial meniscus; ACLT: anterior cruciate ligament transection; MIA: monosodium iodoacetate; FMT: fecal microbiota transplantation; SFB: segmented filamentous bacteria; AP-1: activator protein-1; FGFR3: fibroblast growth factor receptor 3; FTH1: ferritin heavy chain 1; GPX4: glutathione peroxidase 4; NCOA4: nuclear receptor coactivator 4; NLRP3: NOD-like receptor thermal protein domain associated protein 3; NQO1: NAD(P)H: quinone oxidoreductase 1; Osx: osterix; RANKL: receptor activator of nuclear factor-κB ligand; RIP2: receptor-interacting protein 2; ROS: reactive oxygen species; TWEAK: TNF-related weak inducer of apoptosis; UA: urolithin A; UB: urolithin B; CRP: C-reactive protein; MYD88: myeloid differentiation primary response 88.
